# LongITools: Dynamic longitudinal exposome trajectories in cardiovascular and metabolic noncommunicable diseases

**DOI:** 10.1097/EE9.0000000000000184

**Published:** 2021-12-28

**Authors:** Justiina Ronkainen, Rozenn Nedelec, Angelica Atehortua, Zhanna Balkhiyarova, Anna Cascarano, Vien Ngoc Dang, Ahmed Elhakeem, Esther van Enckevort, Ana Goncalves Soares, Sido Haakma, Miia Halonen, Katharina F. Heil, Anni Heiskala, Eleanor Hyde, Bénédicte Jacquemin, Elina Keikkala, Jules Kerckhoffs, Anton Klåvus, Joanna A. Kopinska, Johanna Lepeule, Francesca Marazzi, Irina Motoc, Mari Näätänen, Anton Ribbenstedt, Amanda Rundblad, Otto Savolainen, Valentina Simonetti, Nina de Toro Eadie, Evangelia Tzala, Anna Ulrich, Thomas Wright, Iman Zarei, Enrico d’Amico, Federico Belotti, Carl Brunius, Christopher Castleton, Marie-Aline Charles, Romy Gaillard, Kati Hanhineva, Gerard Hoek, Kirsten B. Holven, Vincent W. V. Jaddoe, Marika A. Kaakinen, Eero Kajantie, Maryam Kavousi, Timo Lakka, Jason Matthews, Andrea Piano Mortari, Marja Vääräsmäki, Trudy Voortman, Claire Webster, Marie Zins, Vincenzo Atella, Maria Bulgheroni, Marc Chadeau-Hyam, Gabriella Conti, Jayne Evans, Janine F. Felix, Barbara Heude, Marjo-Riitta Järvelin, Marjukka Kolehmainen, Rikard Landberg, Karim Lekadir, Stefano Parusso, Inga Prokopenko, Susanne R. de Rooij, Tessa Roseboom, Morris Swertz, Nicholas Timpson, Stine M. Ulven, Roel Vermeulen, Teija Juola, Sylvain Sebert

**Affiliations:** a Center for Life Course Health Research, University of Oulu, Oulu, Finland; b Artificial Intelligence in Medicine Lab (BCN-AIM), University of Barcelona, Barcelona, Spain; c Department of Mathematics and Computer Science, University of Barcelona, Barcelona, Spain; d Section of Statistical Multi-Omics, Department of Clinical and Experimental Medicine, School of Biosciences and Medicine, University of Surrey, Guildford, United Kingdom; e Section of Genetics and Genomics, Department of Metabolism, Digestion and Reproduction, Imperial College London, London, United Kingdom; f Bashkir State Medical University, Department of Endocrinology, Ufa, Russian Federation; g Population Health Sciences, Bristol Medical School, University of Bristol, Bristol, United Kingdom; h MRC Integrative Epidemiology Unit at the University of Bristol, Bristol, United Kingdom; i Department of Genetics and Genomics Coordination Center, University of Groningen, Groningen, the Netherlands; j University of Rennes, INSERM, School of Advanced Studies in Public Health (EHESP), Research Institute for Environmental and Occupational Health, UMR_S 1085, Rennes, France; k Finnish Institute for Health and Welfare, Population Health Unit, Helsinki and Oulu, Finland; l PEDEGO Research Unit, MRC Oulu, Oulu University Hospital and University of Oulu, Oulu, Finland; m Institute for Risk Assessment Sciences, Utrecht University, Utrecht, the Netherlands; n Institute of Public Health and Clinical Nutrition, University of Eastern Finland, Kuopio, Finland; o Department of Social Sciences and Economics, Sapienza University of Rome, Rome, Italy; p Grenoble Alpes University, INSERM, CNRS, Institute for Advanced Biosciences, Grenoble, France; q CEIS Tor Vergata, Centre for Economic and International Studies, University of Rome Tor Vergata, Rome, Italy; r Amsterdam UMC, Epidemiology and Data Science, University of Amsterdam, Amsterdam Public Health, Amsterdam, the Netherlands; s Division of Food and Nutrition Science, Department of Biology and Biological Engineering, Chalmers University of Technology, Gothenburg, Sweden; t Department of Nutrition, Institute of Basic Medical Sciences, University of Oslo, Blindern, Oslo, Norway; u Department of Biology and Biological Engineering, Chalmers Mass Spectrometry Infrastructure, Chalmers University of Technology, Gothenburg, Sweden; v R&D Department, Ab.Acus srl, Milan, Italy; w School of Public Health, Department of Epidemiology and Biostatistics, Imperial College London, St. Mary’s Hospital, London, United Kingdom; x Department of Economics and Finance, University of Rome Tor Vergata, Rome, Italy; y CyNexo srl, Trivignano Udinese, Italy; z Center for Research in Epidemiology and Statistics, INSERM, INRAE, University of Paris, Paris, France; aa Ined, INSERM, EFS, Elfe Joint Unit, Aubervilliers, France; bb The Generation R Study Group, Erasmus MC, University Medical Center Rotterdam, Rotterdam, the Netherlands; cc Department of Pediatrics, Erasmus MC, University Medical Center Rotterdam, Rotterdam, the Netherlands; dd Department of Biochemistry, University of Turku, Turku, Finland; ee National Advisory Unit on Familial Hypercholesterolemia, Department of Endocrinology, Morbid Obesity and Preventive Medicine, Oslo University Hospital, Oslo, Norway; ff Department of Clinical and Molecular Medicine, Norwegian University of Science and Technology, Trondheim, Norway; gg Children’s Hospital, Helsinki University Hospital and University of Helsinki, Helsinki, Finland; hh Department of Epidemiology, Erasmus MC, University Medical Center Rotterdam, Rotterdam, the Netherlands; ii Institute of Biomedicine/Physiology, University of Eastern Finland, Kuopio, Finland; jj Department of Clinical Physiology and Nuclear Medicine, Kuopio University Hospital, Kuopio, Finland; kk Foundation for Research in Health Exercise and Nutrition, Kuopio Research Institute of Exercise Medicine, Kuopio, Finland; ll Beta Technology Ltd, Doncaster, United Kingdom; mm Population-based Epidemiological Cohorts Unit, INSERM UMS 11, Villejuif, France; nn Stanford University, Stanford, CA; oo Department of Economics, University College London, London, United Kingdom; pp Social Research Institute, London, United Kingdom; qq UMR 8199-EGID, Institut Pasteur de Lille, CNRS, University of Lille, Lille, France; rr Institute of Biochemistry and Genetics, Ufa Federal Research Centre Russian Academy of Sciences, Ufa, Russian Federation; ss Gynaecology and Obstetrics, Amsterdam Reproduction and Development Institute, Amsterdam UMC, University of Amsterdam, Amsterdam, the Netherlands; tt Julius Center, University Medical Center Utrecht, Utrecht, the Netherlands; Center for Life Course Health Research, University of Oulu, Oulu, Finland; Center for Life Course Health Research, University of Oulu, Oulu, Finland; Center for Life Course Health Research, University of Oulu, Oulu, Finland; Center for Life Course Health Research, University of Oulu, Oulu, Finland; Center for Life Course Health Research, University of Oulu, Oulu, Finland; Center for Life Course Health Research, University of Oulu, Oulu, Finland; Center for Life Course Health Research, University of Oulu, Oulu, Finland; Center for Life Course Health Research, University of Oulu, Oulu, Finland; , Medical Research Center Oulu, Oulu University Hospital and University of Oulu, Oulu, Finland; Center for Life Course Health Research, University of Oulu, Oulu, Finland; , Research Unit of Mathematical Sciences, University of Oulu, Oulu, Finland; Finnish Institute for Health and Welfare, Population Health Unit, Helsinki and Oulu, Finland; , PEDEGO Research Unit, MRC Oulu, Oulu University Hospital and University of Oulu, Oulu, Finland; , Department of Clinical and Molecular Medicine, Norwegian University of Science and Technology, Trondheim, Norway; , Children’s Hospital, Helsinki University Hospital and University of Helsinki, Helsinki, Finland; Finnish Institute for Health and Welfare, Population Health Unit, Helsinki and Oulu, Finland; , PEDEGO Research Unit, MRC Oulu, Oulu University Hospital and University of Oulu, Oulu, Finland; Finnish Institute for Health and Welfare, Population Health Unit, Helsinki and Oulu, Finland; , PEDEGO Research Unit, MRC Oulu, Oulu University Hospital and University of Oulu, Oulu, Finland; Borealis Biobank of Northern Finland, Oulu University Hospital, Oulu, Finland; Borealis Biobank of Northern Finland, Oulu University Hospital, Oulu, Finland; The Generation R Study Group, Erasmus MC, University Medical Center Rotterdam, Rotterdam, the Netherlands; , Department of Pediatrics, Erasmus MC, University Medical Center Rotterdam, Rotterdam, the Netherlands; The Generation R Study Group, Erasmus MC, University Medical Center Rotterdam, Rotterdam, the Netherlands; , Department of Pediatrics, Erasmus MC, University Medical Center Rotterdam, Rotterdam, the Netherlands; The Generation R Study Group, Erasmus MC, University Medical Center Rotterdam, Rotterdam, the Netherlands; , Department of Pediatrics, Erasmus MC, University Medical Center Rotterdam, Rotterdam, the Netherlands; The Generation R Study Group, Erasmus MC, University Medical Center Rotterdam, Rotterdam, the Netherlands; , Department of Pediatrics, Erasmus MC, University Medical Center Rotterdam, Rotterdam, the Netherlands; Department of Epidemiology, Erasmus MC, University Medical Center Rotterdam, Rotterdam, the Netherlands; Department of Epidemiology, Erasmus MC, University Medical Center Rotterdam, Rotterdam, the Netherlands; Department of Epidemiology, Erasmus MC, University Medical Center Rotterdam, Rotterdam, the Netherlands; School of Public Health, Department of Epidemiology and Biostatistics, Imperial College London, St. Mary’s Hospital, London, United Kingdom; Center for Life Course Health Research, University of Oulu, Oulu, Finland; , School of Public Health, Department of Epidemiology and Biostatistics, Imperial College London, St. Mary’s Hospital, London, United Kingdom; School of Public Health, Department of Epidemiology and Biostatistics, Imperial College London, St. Mary’s Hospital, London, United Kingdom; School of Public Health, Department of Epidemiology and Biostatistics, Imperial College London, St. Mary’s Hospital, London, United Kingdom; School of Public Health, Department of Epidemiology and Biostatistics, Imperial College London, St. Mary’s Hospital, London, United Kingdom; School of Public Health, Department of Epidemiology and Biostatistics, Imperial College London, St. Mary’s Hospital, London, United Kingdom; School of Public Health, Department of Epidemiology and Biostatistics, Imperial College London, St. Mary’s Hospital, London, United Kingdom; Beta Technology Ltd, Doncaster, United Kingdom; Beta Technology Ltd, Doncaster, United Kingdom; Institute of Public Health and Clinical Nutrition, University of Eastern Finland, Kuopio, Finland; Department of Pediatrics, Erasmus MC, University Medical Center Rotterdam, Rotterdam, the Netherlands; , Institute of Public Health and Clinical Nutrition, University of Eastern Finland, Kuopio, Finland; , Department of Biochemistry, University of Turku, Turku, Finland; Institute of Biomedicine/Physiology, University of Eastern Finland, Kuopio, Finland; , Department of Clinical Physiology and Nuclear Medicine, Kuopio University Hospital, Kuopio, Finland; , Foundation for Research in Health Exercise and Nutrition, Kuopio Research Institute of Exercise Medicine, Kuopio, Finland; Institute of Public Health and Clinical Nutrition, University of Eastern Finland, Kuopio, Finland; , Division of Food and Nutrition Science, Department of Biology and Biological Engineering, Chalmers University of Technology, Gothenburg, Sweden; , Department of Biology and Biological Engineering, Chalmers Mass Spectrometry Infrastructure, Chalmers University of Technology, Gothenburg, Sweden; Institute of Public Health and Clinical Nutrition, University of Eastern Finland, Kuopio, Finland; Institute of Public Health and Clinical Nutrition, University of Eastern Finland, Kuopio, Finland; Institute of Public Health and Clinical Nutrition, University of Eastern Finland, Kuopio, Finland; Institute of Public Health and Clinical Nutrition, University of Eastern Finland, Kuopio, Finland; Institute of Biomedicine/Physiology, University of Eastern Finland, Kuopio, Finland; Institute of Public Health and Clinical Nutrition, University of Eastern Finland, Kuopio, Finland; Department of Applied Physics, University of Eastern Finland, Kuopio, Finland; , Department of Environmental and Biological Sciences, University of Eastern Finland, Kuopio, Finland; Institute of Biomedicine/Physiology, University of Eastern Finland, Kuopio, Finland; Institute of Biomedicine/Physiology, University of Eastern Finland, Kuopio, Finland; Division of Food and Nutrition Science, Department of Biology and Biological Engineering, Chalmers University of Technology, Gothenburg, Sweden; Division of Food and Nutrition Science, Department of Biology and Biological Engineering, Chalmers University of Technology, Gothenburg, Sweden; Division of Food and Nutrition Science, Department of Biology and Biological Engineering, Chalmers University of Technology, Gothenburg, Sweden; Department of Genetics and Genomics Coordination Center, University of Groningen, Groningen, the Netherlands; Department of Genetics and Genomics Coordination Center, University of Groningen, Groningen, the Netherlands; Department of Genetics and Genomics Coordination Center, University of Groningen, Groningen, the Netherlands; Department of Genetics and Genomics Coordination Center, University of Groningen, Groningen, the Netherlands; Center for Research in Epidemiology and Statistics, INSERM, INRAE, University of Paris, Paris, France; Center for Research in Epidemiology and Statistics, INSERM, INRAE, University of Paris, Paris, France; , Ined, INSERM, EFS, Elfe Joint Unit, Aubervilliers, France; Population-Based Epidemiological Cohorts Unit, INSERM UMS 11, Villejuif, France; Grenoble Alpes University, INSERM, CNRS, Institute for Advanced Biosciences, Grenoble, France; University of Rennes, INSERM, School of Advanced Studies in Public Health [EHESP], Research Institute for Environmental and Occupational Health, UMR_S 1085, Rennes, France; Center for Research in Epidemiology and Statistics, INSERM, INRAE, University of Paris, Paris, France; Université Paris-Saclay, Université de Paris, UVSQ, Inserm, UMS 011 “Cohortes en Population,” Villejuif, France; Center for Research in Epidemiology and Statistics, INSERM, INRAE, University of Paris, Paris, France; Université Grenoble Alpes, Inserm, CNRS, Team of Environmental Epidemiology Applied to Development and Respiratory Health, Institute for Advanced Biosciences, La Tronche, France; Department of Economics, University College London, London, United Kingdom; , Social Research Institute, London, United Kingdom; Institute for Risk Assessment Sciences, Utrecht University, Utrecht, the Netherlands; , Julius Center, University Medical Center Utrecht, Utrecht, the Netherlands; Institute for Risk Assessment Sciences, Utrecht University, Utrecht, the Netherlands; Institute for Risk Assessment Sciences, Utrecht University, Utrecht, the Netherlands; Section of Statistical Multi-Omics, Department of Clinical and Experimental Medicine, School of Biosciences and Medicine, University of Surrey, Guildford, United Kingdom; , UMR 8199-EGID, Institut Pasteur de Lille, CNRS, University of Lille, Lille, France; , Institute of Biochemistry and Genetics, Ufa Federal Research Centre Russian Academy of Sciences, Ufa, Russian Federation; Section of Statistical Multi-Omics, Department of Clinical and Experimental Medicine, School of Biosciences and Medicine, University of Surrey, Guildford, United Kingdom; , Section of Genetics and Genomics, Department of Metabolism, Digestion and Reproduction, Imperial College London, London, United Kingdom; Section of Statistical Multi-Omics, Department of Clinical and Experimental Medicine, School of Biosciences and Medicine, University of Surrey, Guildford, United Kingdom; , Section of Genetics and Genomics, Department of Metabolism, Digestion and Reproduction, Imperial College London, London, United Kingdom; , Bashkir State Medical University, Department of Endocrinology, Ufa, Russian Federation; Section of Statistical Multi-Omics, Department of Clinical and Experimental Medicine, School of Biosciences and Medicine, University of Surrey, Guildford, United Kingdom; Amsterdam UMC, Epidemiology and Data Science, University of Amsterdam, Amsterdam Public Health, Amsterdam, the Netherlands; , Gynaecology and Obstetrics, Amsterdam Reproduction and Development Institute, Amsterdam UMC, University of Amsterdam, Amsterdam, the Netherlands; Amsterdam UMC, Epidemiology and Data Science, University of Amsterdam, Amsterdam Public Health, Amsterdam, the Netherlands; Amsterdam UMC, Epidemiology and Data Science, University of Amsterdam, Amsterdam Public Health, Amsterdam, the Netherlands; Department of Nutrition, Institute of Basic Medical Sciences, University of Oslo, Blindern, Oslo, Norway; Department of Nutrition, Institute of Basic Medical Sciences, University of Oslo, Blindern, Oslo, Norway; , National Advisory Unit on Familial Hypercholesterolemia, Department of Endocrinology, Morbid Obesity and Preventive Medicine, Oslo University Hospital, Oslo, Norway; Department of Nutrition, Institute of Basic Medical Sciences, University of Oslo, Blindern, Oslo, Norway; Department of Nutrition, Institute of Basic Medical Sciences, University of Oslo, Blindern, Oslo, Norway; Department of Nutrition, Institute of Basic Medical Sciences, University of Oslo, Blindern, Oslo, Norway; Population Health Sciences, Bristol Medical School, University of Bristol, Bristol, United Kingdom; , MRC Integrative Epidemiology Unit at the University of Bristol, Bristol, United Kingdom; Population Health Sciences, Bristol Medical School, University of Bristol, Bristol, United Kingdom; , MRC Integrative Epidemiology Unit at the University of Bristol, Bristol, United Kingdom; Population Health Sciences, Bristol Medical School, University of Bristol, Bristol, United Kingdom; , MRC Integrative Epidemiology Unit at the University of Bristol, Bristol, United Kingdom; Artificial Intelligence in Medicine Lab [BCN-AIM], University of Barcelona, Barcelona, Spain; , Department of Mathematics and Computer Science, University of Barcelona, Barcelona, Spain; Artificial Intelligence in Medicine Lab [BCN-AIM], University of Barcelona, Barcelona, Spain; , Department of Mathematics and Computer Science, University of Barcelona, Barcelona, Spain; Artificial Intelligence in Medicine Lab [BCN-AIM], University of Barcelona, Barcelona, Spain; , Department of Mathematics and Computer Science, University of Barcelona, Barcelona, Spain; Artificial Intelligence in Medicine Lab [BCN-AIM], University of Barcelona, Barcelona, Spain; , Department of Mathematics and Computer Science, University of Barcelona, Barcelona, Spain; Artificial Intelligence in Medicine Lab [BCN-AIM], University of Barcelona, Barcelona, Spain; , Department of Mathematics and Computer Science, University of Barcelona, Barcelona, Spain; Artificial Intelligence in Medicine Lab [BCN-AIM], University of Barcelona, Barcelona, Spain; , Department of Mathematics and Computer Science, University of Barcelona, Barcelona, Spain; Artificial Intelligence in Medicine Lab [BCN-AIM], University of Barcelona, Barcelona, Spain; , Department of Mathematics and Computer Science, University of Barcelona, Barcelona, Spain; R&D Department, Ab.Acus srl, Milan, Italy; R&D Department, Ab.Acus srl, Milan, Italy; R&D Department, Ab.Acus srl, Milan, Italy; R&D Department, Ab.Acus srl, Milan, Italy; CyNexo srl, Trivignano Udinese, Italy; CyNexo srl, Trivignano Udinese, Italy; CyNexo srl, Trivignano Udinese, Italy; CyNexo srl, Trivignano Udinese, Italy; CEIS Tor Vergata, Centre for Economic and International Studies, University of Rome Tor Vergata, Rome, Italy; , Department of Economics and Finance, University of Rome Tor Vergata, Rome, Italy; , Stanford University, Stanford, CA; CEIS Tor Vergata, Centre for Economic and International Studies, University of Rome Tor Vergata, Rome, Italy; Department of Social Sciences and Economics, Sapienza University of Rome, Rome, Italy; CEIS Tor Vergata, Centre for Economic and International Studies, University of Rome Tor Vergata, Rome, Italy; CEIS Tor Vergata, Centre for Economic and International Studies, University of Rome Tor Vergata, Rome, Italy; CEIS Tor Vergata, Centre for Economic and International Studies, University of Rome Tor Vergata, Rome, Italy; , Department of Economics and Finance, University of Rome Tor Vergata, Rome, Italy

**Keywords:** Exposome, Cardio-metabolic and vascular health, Life-course pathways, European research consortium

## Abstract

The current epidemics of cardiovascular and metabolic noncommunicable diseases have emerged alongside dramatic modifications in lifestyle and living environments. These correspond to changes in our “modern” postwar societies globally characterized by rural-to-urban migration, modernization of agricultural practices, and transportation, climate change, and aging. Evidence suggests that these changes are related to each other, although the social and biological mechanisms as well as their interactions have yet to be uncovered. LongITools, as one of the 9 projects included in the European Human Exposome Network, will tackle this environmental health equation linking multidimensional environmental exposures to the occurrence of cardiovascular and metabolic noncommunicable diseases.

**What this study adds**

This consortium profile paper introduces (1) LongITools’ scientific concepts that are primarily based on longitudinal modeling; (2) the metadata for the project; (3) the expected impact of the project; and finally (4) the strengths and challenges of this endeavor.

## Introduction

From one generation to the next, there are vicious circles operating among the rising prevalence of cardiovascular and metabolic noncommunicable diseases (CM-NCDs), social inequality, spiraling health care costs, and varying quality of living environments. If and through which mechanisms these processes relate to each other is probably one of the greatest epidemiological questions of the 21st century. Undeniably, we are facing complex sociodemographic and medical challenges that can be conceptualized as a network of highly correlated determinants and risk factors. These factors in turn influence longitudinal health trajectories, ultimately contributing to the risk of CM-NCDs and the consequent economic burden. LongITools is 1 of the 9 projects included in the European Human Exposome Network (EHEN; www.humanexposome.eu). EHEN is funded by Horizon 2020, the European Union (EU) Framework Programme for Research and Innovation, and it represents the world’s largest network of projects created to study the impact of environmental exposure on human health. Within the EHEN, LongITools’ task is to study the dynamics of environment and cardiometabolic health and develop tools for exposome research. LongITools brings together European longitudinal data, from prospective cohort studies, randomized controlled trials (RCTs), biobanks, and registries, to construct the basis for longitudinal exposome studies. The overarching aim of LongITools is to understand the environmental, biological, and psychosocial dimensions of CM-NCDs, taking life course factors and a longitudinal approach into consideration. LongITools will improve our understanding on how the exposome, i.e., the combined exposures throughout the life course of an individual, contributes to the risk of CM-NCDs.

### LongITools concepts

**Figure 1.**
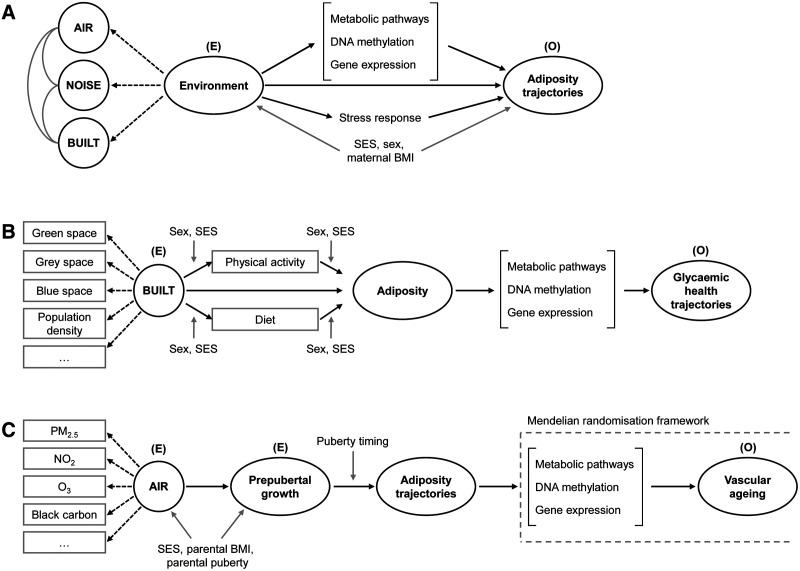
Possible pathways from environment to health outcomes. Directed acyclic graphs representing possible pathways from environmental exposures to adiposity trajectories (A), built environment to glycemic health trajectories (B) and air pollution to vascular aging via growth and adiposity trajectories (C). Black arrows indicate potential causal links, gray arrows indicate confounding paths, and dashed arrows indicate noncausal, latent class paths. AIR indicates air pollution; BMI, body mass index; BUILT, built environment; E, exposure; NOISE, environmental noise; O, outcome; SES, socioeconomic status.

The relationships between environmental risk factors such as air pollution, environmental noise, and urbanization on one hand and the development of CM-NCDs on the other can be conceptualized in several frameworks as exemplified in Figure 1. LongITools is based on a well-established observational relationship between adiposity and increased risk of adverse glycemic, lipid-related, and cardiac functions leading to the development of insulin resistance, hypercholesterolemia, hypertriglyceridemia, and high blood pressure, which in turn together with environmental risk factors are associated with increased risk of CM-NCDs. Various stages of development of CM-NCDs share genetic, biological, lifestyle (unhealthy diet and physical inactivity), environmental, and sociodemographic causes. However, at all stages of the life course, which in LongITools are divided into “early-life,” covering the fetal period to childhood, “adolescence” and “adulthood and old age,” considerable knowledge gaps remain.

Addressing the relationships in Figure 1, LongITools will look to challenge the following assumptions, which are not mutually exclusive, regarding the role of exposome on the life course development of CM-NCDs:

- Direct chain of causality: variations in environmental risk factors are causally related to changes in lipid and glycemic trajectories with different relationships to early disease stages and subsequent development of CM-NCDs. Work within LongITools will attempt to combine data from cohorts as proposed by Hughes et al^1^ and deploy multiple orthogonal analysis designs to challenge the causal chain, such as by implementing cross-cohort comparisons, Mendelian randomization, and Bayesian path models.^2,3^
- Joint effects hypothesis: associations and potentially causal relationships with a single or a set of environmental exposures, reflecting underlying commonality, influence the disease trajectories. This hypothesis can be tested by analyzing how the environmental factors, in isolation or as latent environmental scores, may modify the core relationship between anthropometry, early disease stages, and the onset of CM-NCDs.
- Bidirectional causality hypothesis: the relationships between cardiometabolic health trajectories and onset of CM-NCD promote the deterioration of public health and the environment. The assumption of bidirectionality is assumed correct as mutually promoting risk profiles (i.e., of disease status and environmental exposure) are assessed and potentially demonstrated. In this instance, time series/longitudinal data (with possible crossover events) and bidirectional Mendelian randomization will be used to explore the possible existence of enforcing feed-forward relationships between disease and environment, e.g., morbidity, sociodemographic patterns, and access and exposure profiles to protective or risky environments.
- Critical period hypothesis: there are stages of development within the life course during which environmental risk factors have apparently greater impact on the development of CM-NCDs. In LongITools, we will evaluate the impact of environmental risk factors during the fetal and early childhood period, adolescence, and late adulthood.
- Biological conversion hypothesis: air pollution, climate change, noise, and urbanization can induce biological effects, which persist through the modification of regulatory pathways. LongITools will focus on the possible effects of environmental factors on the changes in DNA methylation (epigenomics), gene expression (transcriptomics), and metabolism (metabolomics).
- Gene-environment hypothesis: genetic variation between individuals may modify the induction of biological effects by environmental factors. This relationship may theoretically occur in reverse, and effort will be put into the examination of apparent interactions, considering challenges in both statistical power and the true origin of apparent interactions.

The source of life course data and the opportunity to make these data findable, accessible, interoperable, and reusable (FAIR) are core components of this consortium.^4^ This consortium profile describes the studies involved in LongITools and the FAIR metadata that the project will build and promote. LongITools is coordinated by the University of Oulu in Finland and includes 15 academic and 3 small- and medium-sized enterprise (SME) partners across Europe (eTable 1; http://links.lww.com/EE/A168). The participants all complement one another, bringing together the full range of technical and specialist expertise in epidemiology, (epi-) genetics, metabolomics, lifestyle, mathematics, economics, policy making, and sensor technology that are required to create a critical mass of expertise for the project.

### Who is in the study?

**Figure 2.**
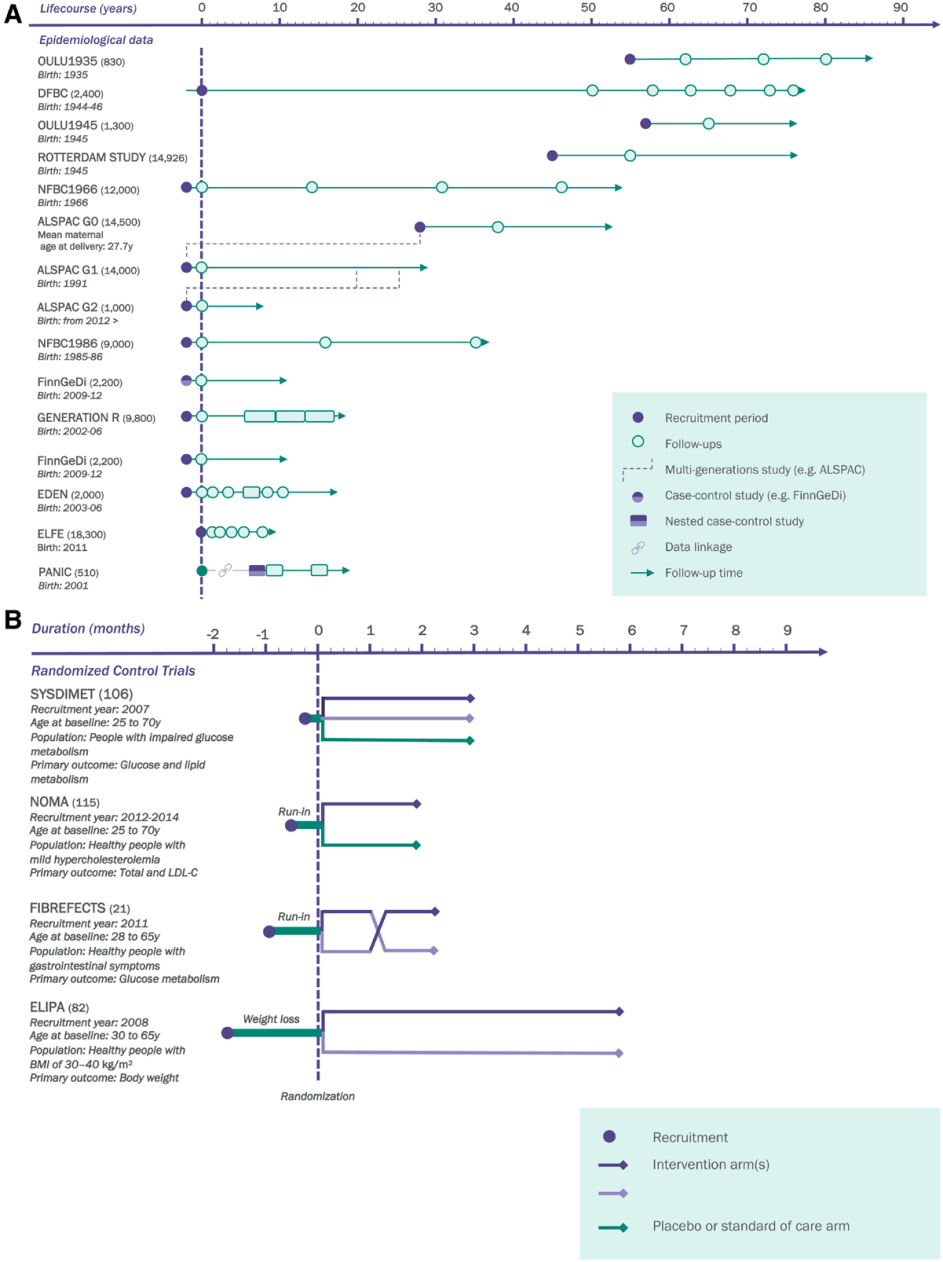
Studies in LongITools. (A) Cohorts and (B) Interventions. ALSPAC indicates Avon Longitudinal Study of Parents and Children; BMI, body mass index; DFBC, Dutch Famine Birth Cohort; EDEN, Etude des Déterminants pré et post natals précoces du développement psychomoteur et de la santé de l’Enfant; ELFE, Etude Longitudinale Française depuis l’Enfance; ELIPA, Foods for Weight Maintenance; Fibrefects, Grain fibre modification for gut-mediated health effects; FinnGeDi, Finnish Gestational Diabetes; LDL-C, low-density lipoprotein cholesterol; NFBC, Northern Finland Birth Cohort; NOMA, Fat Quality on Blood Lipids and Immune Response; OULU1935/45, Born in Oulu in 1935 and 1945; PANIC, Physical Activity and Nutrition in Children; SYSDIMET, Health Grain Intervention.

**Figure 3.**
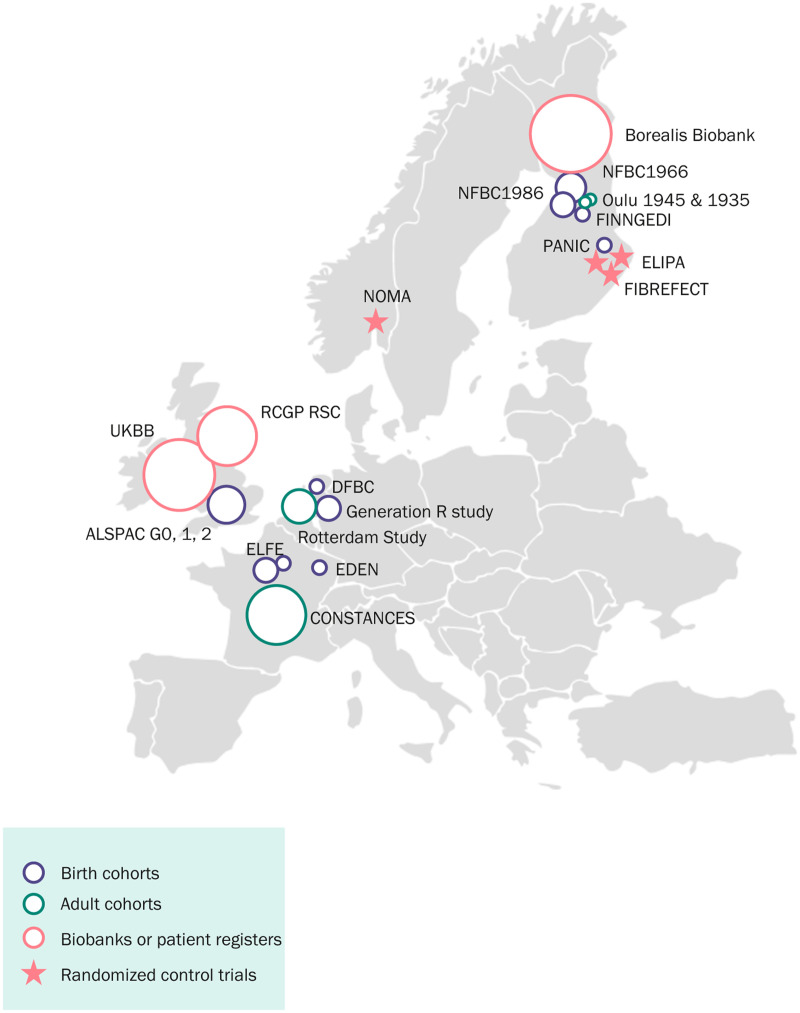
Map of studies participating in LongITools. The size of the circle indicates the relative size of the study. ALSPAC indicates Avon Longitudinal Study of Parents and Children; CONSTANCES, Cohorte des Consultants des Centres d’Examens de Santé; DFBC, Dutch Famine Birth Cohort; EDEN, Etude des Déterminants pré et post natals précoces du développement psychomoteur et de la santé de l’Enfant; ELFE, Etude Longitudinale Française depuis l’Enfance; ELIPA, Foods for Weight Maintenance; Fibrefects, Grain fibre modification for gut-mediated health effects; FinnGeDi, Finnish Gestational Diabetes; NFBC, Northern Finland Birth Cohort; NOMA, Fat Quality on Blood Lipids and Immune Response; OULU1935/45, Born in Oulu in 1935 and 1945; PANIC, Physical Activity and Nutrition in Children; RCGP RSC, Royal College of General Practitioners Research and Surveillance Centre; UKBB, United Kingdom Biobank.

LongITools builds upon and leverages prospective birth cohorts, longitudinal studies in adults, register-based follow-ups, randomized controlled trials (RCTs), patient databases, as well as maternity and hospital biobanks. Currently, these add up to 25 different studies including 11 million individuals across Europe (Table 1). Birth cohorts within the project will provide substantial longitudinal data from pregnancy to adolescence and early adulthood, complemented by prospective adult cohorts, with multiple follow-ups during adulthood and in older age. The RCTs involved are focused on the role of nutrition and physical activity in general health and metabolism. These will not only provide comprehensive biological and exposome profiles of study participants but will also allow an in-depth analysis in more controlled settings. Finally, the involved biobanks will be essential for the generalization of the analyses in large populations. Altogether, the studies involved in LongITools cover the whole life course, represented by blood samples, metadata, and questionnaires of thousands of cohort participants (Figures 2A, B, and 3). The data collected in the studies at different time points are summarized in Table 2 and are available in more detail on the LongITools website (www.longitools.org/about).

**Table 1.** Summary of studies involved in LongITools

Abbreviated study name and reference	Full study name	Study design	Geographic information	Year of establishment*	Sample size at baseline
Prospective birth cohorts					
ALSPAC G0^23^	Avon Longitudinal Study of Parents and Children Generation 0	Prospective adult cohort	Home address geocoded to property and postcode level	1991–1992	14,541
ALSPAC G1^24^	Avon Longitudinal Study of Parents and Children Generation 1	Prospective birth cohort	Home address geocoded to property and postcode level	1991–1992	14,062
ALSPAC G2^25^	Avon Longitudinal Study of Parents and Children Generation 2	Prospective infant/child cohort	Home address geocoded to property and postcode level	2012–2018	850 ongoing
DFBC^26^	Dutch Famine Birth Cohort	Prospective birth cohort	Home address available at each visit	1943	2,414
EDEN^27^	Etude des Déterminants pré et post natals précoces du développement psychomoteur et de la santé de l’Enfant (In French)	Prospective birth cohort	Home address geocoded to property and postcode level	2003	2,002
ELFE^28^	Etude Longitudinale Française depuis l’Enfance (In French)	Prospective birth cohort	Home address available at each visit	2011	18,329
FinnGeDi^29,30^	Finnish Gestational Diabetes Study	Prospective birth cohort	Home address available at baseline	2009–2012	2,212
Generation R^31^	Generation R Study	Prospective birth cohort	Home address available at each visit	2002–2006	9,778
NFBC1966^26^	Northern Finland Birth Cohort 1966	Prospective birth cohort	Home address available from 1966	1966	12,055
NFBC1986^26^	Northern Finland Birth Cohort 1986	Prospective birth cohort	Home address available from 1986	1986	9,432
Prospective adult cohorts					
CONSTANCES^32^	Cohorte des Consultants des Centres d’Examens de Santé (In French)	Prospective adult cohort	Home address available	2012–2019	220,000
RS I^33^	Rotterdam Study, first cohort	Prospective adult cohort	Home address available	1989	7,983
RS II^33^	Rotterdam Study, second cohort	Prospective adult cohort	Home address available	2000	3,011
RS III^33^	Rotterdam Study, third cohort	Prospective adult cohort	Home address available	2006	3,932
OULU1935/45	Born in Oulu in 1935 and 1945	Prospective adult cohort	Home address available	1935–1945	2,000
Interventions and trials					
ELIPA^34^	Elintarvikkeita Painonhallintaan (In Finnish)	RCT	Home address geocoded to property and postcode level	2008	99
Fibrefects^35^	Grain Fibre Modification for Gut-Mediated Health Effects	RCT	Geocoding in process	2011	25
NOMA^36^	Fat Quality on Blood Lipids and Immune Response	RCT	Home address available at baseline	2012–2014	99
PANIC^37^	Physical Activity and Nutrition in Children Study	Controlled intervention	Home address available at baseline	2007–2009	504
SYSDIMET^38^	Health Grain Intervention	RCT	Not available	2007	102
Administrative cohorts and Biobanks					
Borealis Biobank	Borealis Biobank of Northern Finland	Biobank	Home address available	2015†	500,000
FMC	Finnish Maternity Cohort (managed by Borealis Biobank)	Biobank	Home address available upon request	1983	950,000
RCGP RSC	Royal College of General Practitioners Research & Surveillance Centre	Primary care sentinel network	Not available	1990–2018	7,000,000
UK Biobank	United Kingdom Biobank	Biobank	Not available	2006	500,000

*Year of establishment in biobanks corresponds to the year from which samples are available.

†Diagnostic pathology tissue archives starting from 1978 to 8/2013 transferred to the biobank in addition to ongoing prospective biobank consent and sample collection.

RCT indicates randomized controlled trial.

**Table 2.** Data available for general population-based studies and clinical trials in LongITools

Indicator	ALSPAC G0	ALSPAC G1	ALSPAC G2	DFBC	EDEN	ELFE	FinnGeDi	Generation R	NFBC1966	NFBC1986	CONSTANCES	RS	OULU1935/45	ELIPA	Fibrefects	NOMA	PANIC	SYSDIMET
Parental and pregnancy																		
Anthropometric measures	x	x	x	x	x	x	x	x	x	x	-	-	-	-	-	-	x	-
Blood samples	x	x	x	-	x	x	x	x	x	x	-	-	-	-	-	-	-	-
Lifestyle and health behavior	x	x	x	-	x	x	x	x	x	x	-	-	-	-	-	-	x	-
Socioeconomic indicators	x	x	x	x	x	x	x	x	x	x	-	-	-	-	-	-	x	-
GIS/living location	x	x	x	-	x	x	x	x	x	x	-	-	-	-	-	-	x	-
Epigenomics	x	x	-	-	x	-	x	-	-	-	-	-	-	-	-	-	-	-
Transcriptomics	-	x	-	-	-	-	-	-	-	-	-	-	-	-	-	-	-	-
Metabolomics	-	-	-	-	o	-	-	x	-	-	-	-	-	-	-	-	-	-
Childhood																		
Anthropometric measures	-	x	x	-	x	x	x	x	x	x	x	-	-	-	-	-	x	-
Developmental milestones	-	x	x	-	x	x	-	x	x	x	-	-	-	-	-	-	x	-
Growth modeling	-	x	x	-	x	x	-	x	x	x	-	-	-	-	-	-	x	-
Blood samples	-	x	x	-	x	x	-	x	-	-	-	-	-	-	-	-	x	-
Lifestyle and health behavior	-	x	x	-	x	x	-	x	x	x	-	-	-	-	-	-	x	-
Socioeconomic indicators	-	x	x	-	x	x	-	x	x	x	x	-	-	-	-	-	x	-
GIS/living location	-	x	x	-	x	x	-	x	x	x	x	-	-	-	-	-	x	-
Epigenomics	-	x	-	-	x	-	x	x	-	-	-	-	-	-	-	-	-	-
Transcriptomics	-	x	-	-	-	-	-	x	-	-	-	-	-	-	-	-	-	-
Metabolomics	-	x	-	-	o	-	-	o/x	-	-	-	-	-	-	-	-	x	-
Adolescence and early adulthood																		
Anthropometric measures	-	x	-	-	-	-	-	x	x	x	x	-	-	-	-	-	x	-
Blood samples	-	x	-	-	-	-	-	x	x	x	x	-	-	-	-	-	x	-
Lifestyle and health behavior	-	x	-	-	-	-	-	x	x	x	x	-	-	-	-	-	x	-
Socioeconomic indicators	-	x	-	-	-	-	-	x	x	x	x	-	-	-	-	-	x	-
GIS/living location	-	x	-	-	-	-	-	x	x	x	x	-	-	-	-	-	x	-
Epigenomics	-	x	-	-	-	-	-	-	-	x	-	-	-	-	-	-	-	-
Transcriptomics	-	x	-	-	-	-	-	-	-	-	-	-	-	-	-	-	-	-
Metabolomics	-	x	-	-	-	-	-	-	-	x	-	-	-	-	-	-	x	-
Adulthood and old age																		
Anthropometric measures	x	-	-	x	-	-	-	-	x	x	x	x	x	x	x	x	o	x
Blood samples	x	-	-	x	-	-	-	-	x	x	x	x	x	x	x	x	o	x
Lifestyle and health behavior	x	-	-	x	-	-	-	-	x	x	x	x	x	x	x	x	o	x
Socioeconomic indicators	x	-	-	x	-	-	-	-	x	x	x	x	x	x	x	-	o	x
GIS/living location	x	-	-	x	-	-	-	-	x	x	x	x	x	x	x	x	o	-
Epigenomics	x	-	-	x	-	-	-	-	x	-	-	x	-	-	-	-	-	-
Transcriptomics	x	-	-	-	-	-	-	-	-	-	-	x	-	x	-	x	-	x
Metabolomics	x	-	-	x	-	-	-	-	x	-	-	x	-	x	x	x	o	x

- indicates no data collection at this time point; ALSPAC, Avon Longitudinal Study of Parents and Children; CONSTANCES, Cohorte des Consultants des Centres d’Examens de Santé; DFBC, Dutch Famine Birth Cohort; EDEN, Etude des Déterminants pré et post natals précoces du développement psychomoteur et de la santé de l’Enfant; ELFE, Etude Longitudinale Française depuis l’Enfance; ELIPA, Foods for Weight Maintenance; Fibrefects, Grain fibre modification for gut-mediated health effects; FinnGeDi, Finnish Gestational Diabetes; GIS, geographical information system; NFBC, Northern Finland Birth Cohort; NOMA, Fat Quality on Blood Lipids and Immune Response; o, data will be collected during LongITools; OULU1935/45, Born in Oulu in 1935 and 1945; PANIC, Physical Activity and Nutrition in Children; RS, Rotterdam Study; SYSDIMET, Health Grain Intervention; x, data available.

### How do we study?

To optimize findability, all relevant study metadata, i.e., the available variables in the studies, as well as how they are harmonized to be made interoperable for pooled and meta-analysis, will be made findable and accessible into a MOLGENIS catalogue,^5^ linked to the Biobanking and BioMolecular resources Research Infrastructure - European Research Infrastructure Consortium (BBMRI-ERIC) Directory of cohorts and biobanks,^6^ and integrated with the existing EU Child Cohort Network Variable Catalogue (https://catalogue.lifecycle-project.eu/) created by the Horizon 2020–funded LifeCycle project.^7^ LongITools will use recommendations from the LifeCycle project when possible and will establish new harmonization instructions when needed. By using centrally administered instructions for harmonization, LongITools aims to ease the collaboration between studies. As all studies historically have their own design and data collection protocols, harmonization may not always make optimal use of all data available in each study. However, the increased statistical power in the pooled and meta-analyses will be the positive trade-off of possible loss of detail caused by harmonization. LongITools will use a federated data analysis platform, DataSHIELD, which enables the analysis without need to physically transport the data.

#### Federated data analysis approach.

LongITools will use DataSHIELD, when technically, scientifically, and ethically relevant, which was developed as part of the EU-FP7 Biobank Standardisation and Harmonisation for Research Excellence in the European Union Project.^8,9^ DataSHIELD enables researchers to analyze data from partner institutions swiftly and securely, respecting the current national and European data protection regulations. To briefly summarize its use, data holders store individual-level data on their own local data warehouse servers and link to the DataSHIELD client portal using the MOLGENIS Armadillo server (https://github.com/molgenis/molgenis-service-armadillo). The connection between the data warehouse and the client portal is restricted so that only analysis commands can pass through from the client portal to the data server and only nondisclosive summary statistics are sent from the data server to the client portal. In this way, analyses using data from multiple studies can be run from a central analyst’s computer, thus strongly increasing analysis speed and decreasing administrative load and local analyst time. Each study controls permissions to identified researchers within LongITools to use their data in any analysis.

### What has been and will be measured?

#### Environmental exposures.

LongITools will use existing pan-European models for air pollution, noise, and green space as established within European projects, such as the European Study of Cohorts for Air Pollution Effects^10,11^ (www.escapeproject.eu) and the Effects of Low-Level Air Pollution: A Study in Europe^12,13^ (www.elapseproject.eu). Following environmental maps will be linked to the individual residential addresses using a geographical information system:

- Air pollution will be assessed using EU-wide air pollution maps at a fine (100 × 100 m) resolution, which have been developed within the European Study of Cohorts for Air Pollution Effects and Effects of Low-Level Air Pollution: A Study in Europe. These use hybrid land use regression modeling, incorporating surface air quality monitoring, satellite monitoring, chemical transport modeling, and fine scale traffic and land use data;
- Noise estimates will be obtained using harmonized pan-European noise exposure models for traffic noise estimates, extending the existing (metropolitan area) maps to the full European population;
- Green space will be assessed using satellite-based indices of greenness such as the normalized difference vegetation index;
- Built environment will be modeled from the geographical information system and translated into indices of walkability, distances, food and sport outlet density, and accessibility of health care services.

These estimates allow LongITools to compose and study the exposomes throughout the life course. In addition, locally collected exposure data will be applied within RCTs to study the impact of environmental effects and their interaction with intervention target factors in the risk markers of CM-NCD within rather short intervention periods. Although largely available and often collected in a standardized way, the data on environmental exposures have possible intrinsic limitations in terms of (1) availability in historical cohorts such as the Dutch Famine Birth Cohort or the Northern Finland Birth Cohort 1966 and (2) heterogeneity of the source (and/or the effects) between countries. This later limitation will be addressed by studying in detail the structure of the data representing the environmental exposures.

#### Internal exposures.

LongITools will analyze the molecular pathways underlying the associations of environmental exposures and cardiometabolic health trajectories by using repeated measures of the internal exposome.

- Epigenomics will be studied by using DNA methylation, which has been measured by the Illumina Infinium Human Methylation 450K BeadChip and MethylationEPIC BeadChip platforms;
- Transcriptomics measures are based on Illumina or Affymetrix arrays and RNA sequencing. e.g., we will use transcriptome data from the RCTs Foods for Weight Maintenance (ELIPA), Fat Quality on Blood Lipids and Immune Response (NOMA), and Health Grain Intervention (SYSDIMET) to analyze how air pollution, noise, and the build environment may mediate their effect on health via change in specific gene expression;
- Metabolomics will be studied using nuclear magnetic resonance or liquid chromatography mass spectrometry–based platforms with methods enabling coverage of a wide repertoire of both endo- and exogenous metabolite classes including amino acids, bile acids, steroids, various lipid classes, microbiota-produced metabolites, diet-derived compounds, and xenobiotics.^14,15^ Nontargeted metabolic profiling will be used to explore the connections between circulating metabolites and the exposure variables, providing metabolic snapshot of the exposome with unique opportunities for molecular epidemiology.^16,17^ These analyses will result in semiquantitative detection of thousands of metabolite features, of which approximately 1000 will be identified a priori. Unidentified metabolites of interest detected from data analysis^18,19^ will be identified using state-of-the-art tools and pipelines.

Information about the availability of the omics data in each LongITools study can be found in Table 2.

#### Health trajectories.

LongITools will use longitudinal, life course modeling throughout its analyses. LongITools will study how the exposome, linked to geocodes from an individual’s birthplace or residential location, is associated with the following cardiovascular and metabolic health trajectories, i.e., the 4 main outcome phenotypes of LongITools:

- Anthropometric trajectories, identified using height and weight measures in infancy, childhood, and adolescence, by using longitudinal growth data or latent trajectory modeling supported by adiposity milestones, such as adiposity peak, adiposity rebound, body mass index at puberty, and life course body mass index trajectories;
- Glycemic health trajectories, identified using repeated measures of glycemic health, such as fasting glucose, fasting insulin, glycosylated hemoglobin, diabetes diagnosis, and diabetes medications;
- Cardiovascular health trajectories, identified using repeated measures of blood pressure, heart rate, indices of cardiac structure and function, cardiac diagnoses, and cardiovascular medications;
- Lipid-related health trajectories, identified using repeated measures of blood lipids, lipoproteins, and related medications.

#### Economic and policy impact.

LongITools will build a comprehensive data set of policy interventions targeting the exposome and health care, which were implemented in the time span and locations covered by the birth cohorts (Figure 2). The aim is to investigate if and how such policy interventions have affected the insurgency of CM-NCDs, in terms of both health status and economic implications. Furthermore, LongITools will estimate, within an economic life course model of health production, the extent to which the economic burden is due to the external exposome and evaluate policy-relevant “what-if” scenarios using a dynamic microsimulation model, i.e., the Future Elderly Model.^20–22^

#### Knowledge exploitation.

The theoretical framework will be carried out on existing data from the LongITools consortium to train artificial intelligence (AI) algorithms, such as random forests, support vector machines and deep neural networks, which will enable translation of data and knowledge into simple and available predictive tools for scientists, citizens, policy makers, or other end users. For this later part, codesigning activities are currently ongoing with multiple stakeholders, including clinicians, AI technologists, social scientists, and exposome experts, to define the functional and user requirements for these AI-powered digital tools. The steps being developed to achieve this are visualized in eFigure 1; http://links.lww.com/EE/A171 (design and principles of the LongITools health application), here interdisciplinary competences converge. Many variables from environmental and personal domains concur to delineate longitudinal trajectories. Some of them are already available, thanks to digital personal health care devices, while others will be more specific and will need the inclusion of targeted sensors as part of an embedded system (LongIToolsHub). These tools will be validated in a pilot study.

## Strengths and Challenges

LongITools comprises a vast amount of prospective data collected in Europe, harnessed to enhance exposome research, as well as longitudinal and econometric modeling. When combined, these data offer immense potential to inform future European health policy. Furthermore, the data are organized to enable direct replication under the FAIR principles. While sample size allowing statistical power is deemed essential for robust evidence-based strategies, it is also important to combine study designs to validate findings under different statistical assumptions. Another strength of LongITools is the inclusion of data from RCTs for in-depth sensitivity analyses and to identify novel pathways that could be generalized in the cohort setting. Finally, LongITools includes longitudinal birth cohorts and aging cohorts from the same geographical location, which enables us to study the changing environment and its association with cardiovascular and metabolic health.

The key challenge faced by LongITools, and more broadly by all epidemiological study, is to translate the findings into meaningful change for global health. To tackle this, LongITools operates in close collaboration with policy makers throughout the project to convert the results into evidence-based policy options. A critical mass of data and expertise brought together in LongITools offers a substantial resource, which also leads to another challenge faced by the consortium: how to best combine the characteristics of the cohorts involved. The cohorts were established for their own individual purposes before being brought together under this project, and the methods of data collection have thus not been standardized a priori across the consortium. Therefore, consideration is required for the transferability of the statistical models and harmonization of the data. However, this also gives us the opportunity to examine if similar processes operate in different environments and thus to draw conclusions on generalizability. In addition, owing to the internationality of the project, differences in technology, questionnaire data and biospecimen collection methods, terminology and diagnosis definitions, country-specific measurement techniques, and ethical requirements among the studies exist. This heterogeneity can introduce differences in the results between the studies, which can be analyzed when necessary; we can generalize where possible and be specific when needed. In addition, the environmental exposures are harmonized by using the same model, which can also mitigate possible inconsistencies between the studies. The consortium has made significant progress in overcoming these challenges by developing and updating harmonization manual for the key variables and the overarching advantage of LongITools is that all studies provide rich data on similar key exposures and the outcome measures of interest.

## Conclusion

LongITools provides a collection of studies across different time periods and encompassing different life stages, which will enable us to use a life course approach to study the exposome and its role in the trajectories of cardiometabolic health. Valuing the idea of open science, through its innovative data infrastructure, LongITools will spread new knowledge rapidly and efficiently to the other European Human Exposome Network projects and beyond. The generated and combined knowledge can then be used to develop innovative products and services with the potential to create new markets. In this way, LongITools aims to improve EU citizens’ cardiovascular and metabolic health and thereby reduce individual and societal burdens and health care costs of CM-NCDs. Through the cooperation between research teams and SMEs and by using our extensive data, we expect to make several breakthrough discoveries. The evidence-based innovation platform developed in collaboration among academic and industrial partners during the project will support the cross-fertilization of new technologies and stimulate collaborations in developing new products and services within and beyond the European Human Exposome Network. As a proof of concept, LongITools will develop a mobile application for cardiometabolic risk monitoring, combining computational methods to wearable sensors data, realizing effective cooperation between academic and SME partners.

## ACKNOWLEDGMENTS

Cohort- or project-specific acknowledgements are available in eTable 3; http://links.lww.com/EE/A168.

## Conflicts of interest

The authors declare no conflict of interest.

## Supplementary Material



## APPENDIX

LongITools Project Group: Sylvain Sebert (Center for Life Course Health Research, University of Oulu, Oulu, Finland), Teija Juola (Center for Life Course Health Research, University of Oulu, Oulu, Finland), Rozenn Nedelec (Center for Life Course Health Research, University of Oulu, Oulu, Finland), Justiina Ronkainen (Center for Life Course Health Research, University of Oulu, Oulu, Finland), Anni Heiskala (Center for Life Course Health Research, University of Oulu, Oulu, Finland), Miia Halonen (Center for Life Course Health Research, University of Oulu, Oulu, Finland), Yiyan He (Center for Life Course Health Research, University of Oulu, Oulu, Finland), Jouko Miettunen (Center for Life Course Health Research, University of Oulu, Oulu, Finland; , Medical Research Center Oulu, Oulu University Hospital and University of Oulu, Oulu, Finland), Ville Karhunen (Center for Life Course Health Research, University of Oulu, Oulu, Finland; , Research Unit of Mathematical Sciences, University of Oulu, Oulu, Finland), Eero Kajantie (Finnish Institute for Health and Welfare, Population Health Unit, Helsinki and Oulu, Finland; , PEDEGO Research Unit, MRC Oulu, Oulu University Hospital and University of Oulu, Oulu, Finland; , Department of Clinical and Molecular Medicine, Norwegian University of Science and Technology, Trondheim, Norway; , Children’s Hospital, Helsinki University Hospital and University of Helsinki, Helsinki, Finland), Marja Vääräsmäki (Finnish Institute for Health and Welfare, Population Health Unit, Helsinki and Oulu, Finland; , PEDEGO Research Unit, MRC Oulu, Oulu University Hospital and University of Oulu, Oulu, Finland), Elina Keikkala (Finnish Institute for Health and Welfare, Population Health Unit, Helsinki and Oulu, Finland; , PEDEGO Research Unit, MRC Oulu, Oulu University Hospital and University of Oulu, Oulu, Finland), Pia Nyberg (Borealis Biobank of Northern Finland, Oulu University Hospital, Oulu, Finland), Raisa Serpi (Borealis Biobank of Northern Finland, Oulu University Hospital, Oulu, Finland), Janine Felix (The Generation R Study Group, Erasmus MC, University Medical Center Rotterdam, Rotterdam, the Netherlands; , Department of Pediatrics, Erasmus MC, University Medical Center Rotterdam, Rotterdam, the Netherlands), Vincent Jaddoe (The Generation R Study Group, Erasmus MC, University Medical Center Rotterdam, Rotterdam, the Netherlands; , Department of Pediatrics, Erasmus MC, University Medical Center Rotterdam, Rotterdam, the Netherlands), Irene Marques (The Generation R Study Group, Erasmus MC, University Medical Center Rotterdam, Rotterdam, the Netherlands; , Department of Pediatrics, Erasmus MC, University Medical Center Rotterdam, Rotterdam, the Netherlands), Susana Moreira da Silva Santos (The Generation R Study Group, Erasmus MC, University Medical Center Rotterdam, Rotterdam, the Netherlands; , Department of Pediatrics, Erasmus MC, University Medical Center Rotterdam, Rotterdam, the Netherlands), Maryam Kavousi (Department of Epidemiology, Erasmus MC, University Medical Center Rotterdam, Rotterdam, the Netherlands), Trudy Voortman (Department of Epidemiology, Erasmus MC, University Medical Center Rotterdam, Rotterdam, the Netherlands), Bigina Ginos (Department of Epidemiology, Erasmus MC, University Medical Center Rotterdam, Rotterdam, the Netherlands), Marc Chadeau-Hyam (School of Public Health, Department of Epidemiology and Biostatistics, Imperial College London, St. Mary’s Hospital, London, United Kingdom), Marjo-Riitta Järvelin (Center for Life Course Health Research, University of Oulu, Oulu, Finland; , School of Public Health, Department of Epidemiology and Biostatistics, Imperial College London, St. Mary’s Hospital, London, United Kingdom), Evangelia Tzala (School of Public Health, Department of Epidemiology and Biostatistics, Imperial College London, St. Mary’s Hospital, London, United Kingdom), Nina De Toro (School of Public Health, Department of Epidemiology and Biostatistics, Imperial College London, St. Mary’s Hospital, London, United Kingdom), Thomas Wright (School of Public Health, Department of Epidemiology and Biostatistics, Imperial College London, St. Mary’s Hospital, London, United Kingdom), Barbara Bodinier (School of Public Health, Department of Epidemiology and Biostatistics, Imperial College London, St. Mary’s Hospital, London, United Kingdom), Sonia Dagnino (School of Public Health, Department of Epidemiology and Biostatistics, Imperial College London, St. Mary’s Hospital, London, United Kingdom), Jayne Evans (Beta Technology Ltd, Doncaster, United Kingdom), Claire Webster (Beta Technology Ltd, Doncaster, United Kingdom), Marjukka Kolehmainen (Institute of Public Health and Clinical Nutrition, University of Eastern Finland, Kuopio, Finland), Kati Hanhineva (Department of Pediatrics, Erasmus MC, University Medical Center Rotterdam, Rotterdam, the Netherlands; , Institute of Public Health and Clinical Nutrition, University of Eastern Finland, Kuopio, Finland; , Department of Biochemistry, University of Turku, Turku, Finland), Timo Lakka (Institute of Biomedicine/Physiology, University of Eastern Finland, Kuopio, Finland; , Department of Clinical Physiology and Nuclear Medicine, Kuopio University Hospital, Kuopio, Finland; , Foundation for Research in Health Exercise and Nutrition, Kuopio Research Institute of Exercise Medicine, Kuopio, Finland), Otto Savolainen (Institute of Public Health and Clinical Nutrition, University of Eastern Finland, Kuopio, Finland; , Division of Food and Nutrition Science, Department of Biology and Biological Engineering, Chalmers University of Technology, Gothenburg, Sweden; , Department of Biology and Biological Engineering, Chalmers Mass Spectrometry Infrastructure, Chalmers University of Technology, Gothenburg, Sweden), Iman Zarei (Institute of Public Health and Clinical Nutrition, University of Eastern Finland, Kuopio, Finland), Mari Näätänen (Institute of Public Health and Clinical Nutrition, University of Eastern Finland, Kuopio, Finland), Anton Klåvus (Institute of Public Health and Clinical Nutrition, University of Eastern Finland, Kuopio, Finland), Aino-Maija Eloranta (Institute of Public Health and Clinical Nutrition, University of Eastern Finland, Kuopio, Finland), Juuso Väistö (Institute of Biomedicine/Physiology, University of Eastern Finland, Kuopio, Finland), Anna Kårlund (Institute of Public Health and Clinical Nutrition, University of Eastern Finland, Kuopio, Finland), Santtu Mikkonen (Department of Applied Physics, University of Eastern Finland, Kuopio, Finland; , Department of Environmental and Biological Sciences, University of Eastern Finland, Kuopio, Finland), Merja Atalay (Institute of Biomedicine/Physiology, University of Eastern Finland, Kuopio, Finland), Mustafa Atalay (Institute of Biomedicine/Physiology, University of Eastern Finland, Kuopio, Finland), Rikard Landberg (Division of Food and Nutrition Science, Department of Biology and Biological Engineering, Chalmers University of Technology, Gothenburg, Sweden), Carl Brunius (Division of Food and Nutrition Science, Department of Biology and Biological Engineering, Chalmers University of Technology, Gothenburg, Sweden), Anton Ribbenstedt (Division of Food and Nutrition Science, Department of Biology and Biological Engineering, Chalmers University of Technology, Gothenburg, Sweden), Morris Swertz (Department of Genetics and Genomics Coordination Center, University of Groningen, Groningen, the Netherlands), Eleanor Hyde (Department of Genetics and Genomics Coordination Center, University of Groningen, Groningen, the Netherlands), Sido Haakma (Department of Genetics and Genomics Coordination Center, University of Groningen, Groningen, the Netherlands), Esther van Enckevort (Department of Genetics and Genomics Coordination Center, University of Groningen, Groningen, the Netherlands), Barbara Heude (Center for Research in Epidemiology and Statistics, INSERM, INRAE, University of Paris, Paris, France), Marie-Aline Charles (Center for Research in Epidemiology and Statistics, INSERM, INRAE, University of Paris, Paris, France; , Ined, INSERM, EFS, Elfe Joint Unit, Aubervilliers, France), Marie Zins (Population-Based Epidemiological Cohorts Unit, INSERM UMS 11, Villejuif, France), Johanna Lepeule (Grenoble Alpes University, INSERM, CNRS, Institute for Advanced Biosciences, Grenoble, France), Bénédicte Jacquemin (University of Rennes, INSERM, School of Advanced Studies in Public Health [EHESP], Research Institute for Environmental and Occupational Health, UMR_S 1085, Rennes, France), Lucinda Calas (Center for Research in Epidemiology and Statistics, INSERM, INRAE, University of Paris, Paris, France), Emeline Lequy-Flahault (Université Paris-Saclay, Université de Paris, UVSQ, Inserm, UMS 011 “Cohortes en Population,” Villejuif, France), Wen Lun Yuan (Center for Research in Epidemiology and Statistics, INSERM, INRAE, University of Paris, Paris, France), Emie Seyve (Université Grenoble Alpes, Inserm, CNRS, Team of Environmental Epidemiology Applied to Development and Respiratory Health, Institute for Advanced Biosciences, La Tronche, France), Gabriella Conti (Department of Economics, University College London, London, United Kingdom; , Social Research Institute, London, United Kingdom), Roel Vermeulen (Institute for Risk Assessment Sciences, Utrecht University, Utrecht, the Netherlands; , Julius Center, University Medical Center Utrecht, Utrecht, the Netherlands), Gerard Hoek (Institute for Risk Assessment Sciences, Utrecht University, Utrecht, the Netherlands), Jules Kerckhoffs (Institute for Risk Assessment Sciences, Utrecht University, Utrecht, the Netherlands), Inga Prokopenko (Section of Statistical Multi-Omics, Department of Clinical and Experimental Medicine, School of Biosciences and Medicine, University of Surrey, Guildford, United Kingdom; , UMR 8199-EGID, Institut Pasteur de Lille, CNRS, University of Lille, Lille, France; , Institute of Biochemistry and Genetics, Ufa Federal Research Centre Russian Academy of Sciences, Ufa, Russian Federation), Marika Kaakinen (Section of Statistical Multi-Omics, Department of Clinical and Experimental Medicine, School of Biosciences and Medicine, University of Surrey, Guildford, United Kingdom; , Section of Genetics and Genomics, Department of Metabolism, Digestion and Reproduction, Imperial College London, London, United Kingdom), Zhanna Balkhiyarova (Section of Statistical Multi-Omics, Department of Clinical and Experimental Medicine, School of Biosciences and Medicine, University of Surrey, Guildford, United Kingdom; , Section of Genetics and Genomics, Department of Metabolism, Digestion and Reproduction, Imperial College London, London, United Kingdom; , Bashkir State Medical University, Department of Endocrinology, Ufa, Russian Federation), Anna Ulrich (Section of Statistical Multi-Omics, Department of Clinical and Experimental Medicine, School of Biosciences and Medicine, University of Surrey, Guildford, United Kingdom), Tessa Roseboom (Amsterdam UMC, Epidemiology and Data Science, University of Amsterdam, Amsterdam Public Health, Amsterdam, the Netherlands; , Gynaecology and Obstetrics, Amsterdam Reproduction and Development Institute, Amsterdam UMC, University of Amsterdam, Amsterdam, the Netherlands), Susanne De Rooij (Amsterdam UMC, Epidemiology and Data Science, University of Amsterdam, Amsterdam Public Health, Amsterdam, the Netherlands), Irina Motoc (Amsterdam UMC, Epidemiology and Data Science, University of Amsterdam, Amsterdam Public Health, Amsterdam, the Netherlands), Stine Marie Ulven (Department of Nutrition, Institute of Basic Medical Sciences, University of Oslo, Blindern, Oslo, Norway), Kirsten B. Holven (Department of Nutrition, Institute of Basic Medical Sciences, University of Oslo, Blindern, Oslo, Norway; , National Advisory Unit on Familial Hypercholesterolemia, Department of Endocrinology, Morbid Obesity and Preventive Medicine, Oslo University Hospital, Oslo, Norway), Jason Matthews (Department of Nutrition, Institute of Basic Medical Sciences, University of Oslo, Blindern, Oslo, Norway), Amanda Rundblad (Department of Nutrition, Institute of Basic Medical Sciences, University of Oslo, Blindern, Oslo, Norway), Siddhartha Das (Department of Nutrition, Institute of Basic Medical Sciences, University of Oslo, Blindern, Oslo, Norway), Nicholas Timpson (Population Health Sciences, Bristol Medical School, University of Bristol, Bristol, United Kingdom; , MRC Integrative Epidemiology Unit at the University of Bristol, Bristol, United Kingdom), Ahmed Elhakeem (Population Health Sciences, Bristol Medical School, University of Bristol, Bristol, United Kingdom; , MRC Integrative Epidemiology Unit at the University of Bristol, Bristol, United Kingdom), Ana Luiza Goncalves Soares (Population Health Sciences, Bristol Medical School, University of Bristol, Bristol, United Kingdom; , MRC Integrative Epidemiology Unit at the University of Bristol, Bristol, United Kingdom), Karim Lekadir (Artificial Intelligence in Medicine Lab [BCN-AIM], University of Barcelona, Barcelona, Spain; , Department of Mathematics and Computer Science, University of Barcelona, Barcelona, Spain), Anna Maria Cascarano (Artificial Intelligence in Medicine Lab [BCN-AIM], University of Barcelona, Barcelona, Spain; , Department of Mathematics and Computer Science, University of Barcelona, Barcelona, Spain), Angélica Maria Atehortua Labrador (Artificial Intelligence in Medicine Lab [BCN-AIM], University of Barcelona, Barcelona, Spain; , Department of Mathematics and Computer Science, University of Barcelona, Barcelona, Spain), Vien Ngoc Dang (Artificial Intelligence in Medicine Lab [BCN-AIM], University of Barcelona, Barcelona, Spain; , Department of Mathematics and Computer Science, University of Barcelona, Barcelona, Spain), Katharina F. Heil (Artificial Intelligence in Medicine Lab [BCN-AIM], University of Barcelona, Barcelona, Spain; , Department of Mathematics and Computer Science, University of Barcelona, Barcelona, Spain), Catherine Gallin (Artificial Intelligence in Medicine Lab [BCN-AIM], University of Barcelona, Barcelona, Spain; , Department of Mathematics and Computer Science, University of Barcelona, Barcelona, Spain), Oliver Díaz (Artificial Intelligence in Medicine Lab [BCN-AIM], University of Barcelona, Barcelona, Spain; , Department of Mathematics and Computer Science, University of Barcelona, Barcelona, Spain), Maria Bulgheroni (R&D Department, Ab.Acus srl, Milan, Italy), Valentina Simonetti (R&D Department, Ab.Acus srl, Milan, Italy), Enrico D’Amico (R&D Department, Ab.Acus srl, Milan, Italy), Laura Giani (R&D Department, Ab.Acus srl, Milan, Italy), Fabrizio Manzino (CyNexo srl, Trivignano Udinese, Italy), Stefano Parusso (CyNexo srl, Trivignano Udinese, Italy), Christopher Castleton (CyNexo srl, Trivignano Udinese, Italy), Diego Moimas (CyNexo srl, Trivignano Udinese, Italy), Vincenzo Atella (CEIS Tor Vergata, Centre for Economic and International Studies, University of Rome Tor Vergata, Rome, Italy; , Department of Economics and Finance, University of Rome Tor Vergata, Rome, Italy; , Stanford University, Stanford, CA), Andrea Piano Mortari (CEIS Tor Vergata, Centre for Economic and International Studies, University of Rome Tor Vergata, Rome, Italy), Joanna Kopinska (Department of Social Sciences and Economics, Sapienza University of Rome, Rome, Italy), Francesca Marazzi (CEIS Tor Vergata, Centre for Economic and International Studies, University of Rome Tor Vergata, Rome, Italy), Matilde Giaccherini (CEIS Tor Vergata, Centre for Economic and International Studies, University of Rome Tor Vergata, Rome, Italy), Federico Belotti (CEIS Tor Vergata, Centre for Economic and International Studies, University of Rome Tor Vergata, Rome, Italy; , Department of Economics and Finance, University of Rome Tor Vergata, Rome, Italy).
