# Supplementary material for: LongITools: Dynamic longitudinal exposome trajectories in cardiovascular and metabolic noncommunicable diseases
Source: Environ Epidemiol. 2021 Dec 28;6(1):e184. doi: 10.1097/EE9.0000000000000184 (PMC8835657; doi:10.1097/EE9.0000000000000184)
Supplement: Supplementary file 1 [file ee9-6-e184-s001.docx]

**LongITools: Dynamic longitudinal exposome trajectories in cardiovascular and metabolic non-communicable diseases**

**Supplementary materials**

Supplementary Table 1: Partners in the LongITools project

| Partners | Country | Type of organisation |
| --- | --- | --- |
| Ab.Acus | Italy | SME |
| Amsterdam UMC - Location AMC | The Netherlands | University |
| Beta Technology | United Kingdom | SME |
| Chalmers University of Technology | Sweden | University |
| Cynexo | Italy | SME |
| Erasmus MC, UMC Rotterdam | The Netherlands | University |
| Imperial College London | United Kingdom | University |
| National Institute of Health and Medical Research (INSERM) | France | Research Institute |
| University College London | United Kingdom | University |
| University Medical Center Groningen | The Netherlands | University |
| University of Barcelona | Spain | University |
| University of Bristol | United Kingdom | University |
| University of Eastern Finland | Finland | University |
| University of Oslo | Norway | University |
| University of Surrey | United Kingdom | University |
| University of Oulu | Finland | University |
| University of Rome Tor Vergata | Italy | University |
| Utrecht University | The Netherlands | University |

Supplementary Table 2: Contact information of the Studies involved in LongITools

| Study name | Full name | Contact partner | PI contact email |
| --- | --- | --- | --- |
| ALSPAC | Avon Longitudinal Study of Parents and Children | University of Bristol | n.j.timpson@bristol.ac.uk |
| Biobank Borealis | Biobank Borealis of Northern Finland | Oulu University Hospital | BiopankkiBorealis@ppshp.fi |
| CONSTANCES | Cohorte des Consultants des Centres d’Examens de Santé | National Institute of Health and Medical Research (INSERM) | [contact@constances.fr](mailto:contact@constances.fr) |
| DFBC | Dutch Famine Birth Cohort | Amsterdam UMC, location AMC | s.r.derooij@amsterdamumc.nl |
| EDEN | Etude des Déterminants pré et post natals précoces du développement psychomoteur et de la santé de l’Enfant | National Institute of Health and Medical Research (INSERM) | etude.eden@inserm.fr |
| ELFE | Etude Longitudinale Française depuis l’Enfance | National Institute of Health and Medical Research (INSERM) | communaute@elfe-france.fr |
| ELIPA | Foods for weight maintenance | University of Eastern Finland | marjukka.kolehmainen@uef.fi |
| Fibrefects | Grain fibre modification for gut-mediated health effects | University of Eastern Finland | marjukka.kolehmainen@uef.fi |
| FinnGeDi | Finnish Gestational Diabetes study | University of Oulu | marja.vaarasmaki@oulu.fi |
| FMC | Finnish Maternity Cohort | Oulu University Hospital | BiopankkiBorealis@ppshp.fi |
| Generation R | Generation Rotterdam | Erasmus MC, University Medical Center Rotterdam | generationr@erasmusmc.nl |
| NFBC1966 | Northern Finland Birth Cohort 1966 | University of Oulu | NFBCprojectcenter@oulu.fi |
| NFBC1986 | Northern Finland Birth Cohort 1986 | University of Oulu | NFBCprojectcenter@oulu.fi |
| NOMA | Fat Quality on Blood Lipids and Immune Response | University of Oslo | smulven@medisin.uio.no |
| OULU 1935 | Born in Oulu in 1935 | University of Oulu | NFBCprojectcenter@oulu.fi |
| OULU 1945 | Born in Oulu in 1945 | University of Oulu | NFBCprojectcenter@oulu.fi |
| PANIC | Physical Activity and Nutrition in Children | University of Eastern Finland | timo.lakka@uef.fi |
| RCGP RSC | Royal College of General Practitioners Research & Surveillance Centre | University of Surrey | i.prokopenko@surrey.ac.uk |
| RS | Rotterdam Study | Erasmus MC, University Medical Center Rotterdam (ERASMUS) | secretariat.epi@erasmusmc.nl |
| SYSDIMET | Health Grain intervention | University of Eastern Finland (UEF) | marjukka.kolehmainen@uef.fi |
| UK biobank | United Kingdom Biobank | University of Surrey (SURREY) | i.prokopenko@surrey.ac.uk |

Supplementary Table 3: Cohort-specific acknowledgment, funding statements and data availability statement.

| Study name | Acknowledgment | Funding | Data availability |
| --- | --- | --- | --- |
| ALSPAC | We are extremely grateful to all the families who took part in this study, the midwives for their help in recruiting them and the whole ALSPAC team, which includes interviewers, computer and laboratory technicians, clerical workers, research scientists, volunteers, managers, receptionists and nurses. | The UK Medical Research Council and Wellcome (Grant ref: 217065/Z/19/Z) and the University of Bristol provide core support for ALSPAC. A comprehensive list of grants funding is available on the ALSPAC website (http://www.bristol.ac.uk/alspac/external/documents/grant317acknowledgements.pdf) | Researchers wanting to use ALSPAC data must complete a research proposal form describing the proposed collaboration and send it to the ALSPAC Executive (alspac-exec@bris.ac.uk). More detail on the process of accessing the data can be found on the study website http://www.bristol.ac.uk/alspac/researchers/access/ |
| Biobank Borealis | Biobank Borealis is most grateful to all who have given the biobank to consent, as well as to the medical, nursing and administrative staff at the hospitals. | Biobank Borealis is supported by the Northern Ostrobothnia Hospital District, the University of Oulu, NordLab and the hospital/healthcare districts of Lapland, Länsi-Pohja, Central Ostrobothnia and Kainuu), Academy of Finland (320001, 309276), BusinessFinland (4685/31/2016), Oulu University Hospital state support for research VTR-funding (K10758), Oulu University research funding Terttu, Horizon2020-funding HEAP (874662) | The Biobank Borealis of Northern Finland can assign samples and related data only for high-quality health sciences research and development. A scientific committee will evaluate all sample and data requests. You can make a feasibility and sample and data request by using FINGENIOUS portal https://site.fingenious.fi/en/. You can also contact Biobank Borealis directly, BiopankkiBorealis (at) ppshp.fi and visit the website www.ppshp.fi/biobankki. |
| CONSTANCES | The authors also gratefully acknowledge the major contribution to the protocol of numerous colleagues, in France and abroad, who helped in the general design of the cohort, and of the participating HSCs. The authors express also their thanks to Dominique Polton from the CNAMTS for her help, and to Christophe Albert and Joël Brulard for the drawing of eligible persons and the access to the CNAV database. | The CONSTANCES cohort is supported by the Caisse Nationale d'Assurance Maladie des travailleurs salariés-CNAMTS, and was funded in its pilot phase by the *Direction générale de la santé”* of the Ministry of Health (CPO 2007–2009), and by the *Institut de Recherche en Santé Publique-Institut Thématique Santé Publique*, and the following sponsors : *Ministère de la santé et des sports, Ministère délégué à la recherche, Institut national de la santé et de la recherche médicale, Institut national du cancer et Caisse nationale de solidarité pour l'autonomie* (AMC10003LSA). CONSTANCES is accredited as a ‘National Infrastructure for Biology and health’ by the governmental *Investissements d'avenir* programme and was funded by the *Agence nationale de la recherche* (ANR-11-INBS-0002 grant). CONSTANCES also receives funding from MSD, AstraZeneca and Lundbeck managed by INSERM-Transfert. CONSTANCES is conducted in partnership with the National Health Insurance Fund administered by CNAMTS, and with the National Retirement Insurance Fund administered by the *Caisse nationale d'assurance vieillesse-CNAV*. Quality control procedures are taken in charge by ClinSearch for the data collected in the HSCs, and by Asqualab and EuroCell for the biological data. | Every group in France or in other countries is entitled to apply to develop a nested project within CONSTANCES and to access to its database. Projects are evaluated twice a year by the CONSTANCES Scientific Committee on feasibility and scientific quality criteria. A Charter describes the rules that have been established for using the CONSTANCES infrastructure. The material needed for applying can be downloaded from the CONSTANCES website: http://www.constances.fr/index_EN.php#propose. |
| DFBC | The authors thank the participants of the DFBC cohort for their willing cooperation. | The DFBC study was supported by  the Diabetes Fonds (Netherlands), the Netherlands Heart Foundation, The European Science Foundation (EUROSTRESS), the European Commission (Brainage, Dynahealth, Longitools), Wellbeing (UK), the Medical Research Council (UK), the Dutch Research Council (NWO Aspasia) and the Academic Medical Centre (Amsterdam, The Netherlands). | Any researcher interested in studying the DFBC data should contact Co-PI Susanne R. de Rooij [s.r.derooij@amsterdamumc.nl]. |
| EDEN | The authors thank the EDEN mother-child cohort study group, whose members are I. Annesi-Maesano, J.Y. Bernard, J. Botton, M.A. Charles, P. Dargent-Molina, B. de Lauzon-Guillain, P. Ducimetière, M. de Agostini, B. Foliguet, A. Forhan, X. Fritel, A. Germa, V. Goua, R. Hankard, B. Heude, M. Kaminski, B. Larroquey, N. Lelong, J. Lepeule, G. Magnin, L. Marchand, C. Nabet, F Pierre, R. Slama, M.J. Saurel-Cubizolles, M. Schweitzer, and O. Thiebaugeorges. | The EDEN study was supported by Foundation for medical research (FRM), National Agency for Research (ANR), National Institute for Research in Public health (IRESP: TGIR cohorte santé 2008 program), French Ministry of Health (DGS), French Ministry of Research, INSERM Bone and Joint Diseases National Research (PRO-A), and Human Nutrition National Research Programs, Paris-Sud University, Nestlé, French National Institute for Population Health Surveillance (InVS), French National Institute for Health Education (INPES), the European Union FP7 programmes (FP7/2007–2013, HELIX, ESCAPE, ENRIECO, Medall projects), Diabetes National Research Program (through a collaboration with the French Association of Diabetic Patients (AFD)), French Agency for Environmental Health Safety (now ANSES), Mutuelle Générale de l’Education Nationale a complementary health insurance (MGEN), French national agency for food security, French-speaking association for the study of diabetes and metabolism (ALFEDIAM). | Any researcher interested in exploring EDEN data should contact directly the corresponding author [barbara.heude@inserm.fr] and [etude.eden@inserm.fr] to complete a dedicated project form for evaluation by the EDEN steering committee. |
| ELFE | We are grateful to: (i) the former members of the ELFE unit, without whom the project would never have started: Henri Léridon, initiator and former PI of the project, Stéphanie Vandentorren, Claudine Pirus and Ando Rakotonirina; (ii) for their expertise and assistance, members of the unit for support functions, notably: Catherine Guevel, Sophie de Visme, Laure Gravier, Meryem Zoubiri, Heélène Juillard, Cédric Ricourt and Adriana Candea; (iii) all the researchers who contribute to the projects as members of the ELFE thematic groups and particularly their coordinators; (iv) all the field research assistants and interviewers; and (v) above all, all the ELFE families who have placed their confidence in us and given up their time to the study. | This work was supported by the: National Research Agency Investment for the Future programme (ANR-11-EQPX-0038); French National Institute for Research in Public Health (IRESP TGIR 2009– 01 programme); Ministry of Higher Education and Research; Ministry of Environment; Ministry of Health; French Agency for Public Health; Ministry of Culture; and National Family Allowance Fund. | The ELFE has an open-data policy after an 18-month exclusivity period for the ELFE-associated research teams following each release of new data. Study protocols, questionnaires and the data catalogue can be found online. Data access requests can also be submitted via the data access platform for approval by the ELFE data-access committee. All proposals must comply with the ELFE data-access policy that can be downloaded from the data-access platform https://pandora.vjf.inserm.fr/public/. |
| ELIPA | The authors gratefully acknowledge Eeva Lajunen for her excellent laboratory assistance and authorized nutritionists M.S. Anne Jaaskelainen M.S. Tarja Martikainen, M.S. Reetta Mustonen, and M.S. Taisa Venalainen for theirs excellent assistance in nutritional and other practical duties throughout the intervention study. The skillful assistance of technicians Pirkko Nousiainen, Heidi Eriksson, and Ulla Osterlund and M.S. Nora Ohls in conducting the satiety tests at VTT is also greatly appreciated. | This work was supported by the Finnish Funding Agency for Technology and Innovation (Tekes, grant 40100/07) and Finnish food manufacturers (Atria Plc, Fazer Group Ltd, Arla Ingman Ltd, Valio Ltd, Sinebrychoff Ltd, Vaasan Ltd, Foodfiles Ltd, Leiras Finland Ltd) and partly by the SalWe Research Program for Mind and Body (Tekes grant 1104/10). | Any researcher interested in studying the ELIPA data should contact the researchers. Please send an email to marjukka.kolehmainen@uef.fi |
| Fibrefects | Authors thank Erja Kinnunen, Eeva Lajunen and Lilli Lotvonen for their excellent technical assistance during the intervention. | This study was carried out as a part of the FIBREFECTS (Grain fiber modification for gut mediated health effects) funded mainly by TEKES – The Finnish Funding Agency for  Technology and Innovation – and to a smaller extent by Oy Karl Fazer Ab, Vaasan Oy, and Ravintoraisio Oy. The study was supported by a grant from Raisio Plc Research Foundation to J. Lappi, Academy of Finland to K. Poutanen, the Nordic Centre of Excellence on ‘Systems biology in controlled dietary interventions and cohort studies’ (SYSDIET; 070014) to M. Kolehmainen, the Nordic Centre of Excellence on ‘Nordic health – whole grain food (HELGA; 070015) to H. Mykkänen, and the Danish Strategic  Research Council, ‘Concepts for enhanced butyrate production to improve colonic health and insulin sensitivity’ (ButCoIns; 10-093526) to KE. Bach Knudsen. | Any researcher interested in studying the FibrEfects data should contact the researchers. Please send an email to marjukka.kolehmainen@uef.fi |
| FinnGeDi | We warmly thank all families for participating and research doctors, midwifes and other personnel in the study hospitals and in National Institute for Health and Welfare for their contribution to prepare and implement FinnGeDi project. | Academy of Finland; Diabetes Research Foundation; Foundation for Pediatric Research; Juho Vainio Foundation; Novo Nordisk Foundation; Signe and Ane Gyllenberg Foundation; Sigrid Jusélius Foundation; Yrjö Jahnsson Foundation; Finnish Medical Foundation; Research Funds of Oulu University Hospital (state grants), Research Funds of Oulu University Hospital and Helsinki University Hospital (state grants), Medical Research Center Oulu and Finnish Institute for Health and Welfare. | Access to clinical data is regulated by ethics approvals and individual consent. Access to registry data is subject to permission from the registry authorities. For inquiries regarding possible collaboration, please contact FinnGeDi’s principal investigator and study coordinator, professor, Marja Vääräsmäki, MD, PhD: marja.vaarasmaki@oulu.fi |
| Generation R | The Generation R Study is conducted by the Erasmus MC, University Medical Center Rotterdam in close collaboration with the School of Law and Faculty of Social Sciences of the Erasmus University Rotterdam, the Municipal Health Service Rotterdam area, Rotterdam, the Rotterdam Homecare Foundation, Rotterdam and the Stichting Trombosedienst & Artsenlaboratorium Rijnmond (STAR-MDC), Rotterdam. We gratefully acknowledge the contribution of children and parents, general practitioners, hospitals, midwives and pharmacies in Rotterdam. | The general design of the Generation R Study is made possible by financial support from the Erasmus MC, University Medical Center Rotterdam, Erasmus University Rotterdam, the Netherlands Organization for Health Research and Development and the Ministry of Health, Welfare and Sport. | Requests for access to the Generation R Study data can be addressed to the Director of the Core Facility Generation R, Prof. Dr. Vincent Jaddoe, via generationr@erasmusmc.nl. Requests will be evaluated by the Generation R Management Team taking into account local rules and regulations. |
| NFBC1966 | We thank all cohort members and researchers who participated in the study. We also wish to acknowledge the work of the NFBC project center. | NFNC1966 received financial support from the University of Oulu Grant no. 65354 and 24000692, Oulu University Hospital Grant no. 2/97, 8/97 and 24301140, Ministry of Health and Social Affairs Grant no. 23/251/97, 160/97, 190/97, National Institute for Health and Welfare, Helsinki Grant no. 54121, Regional Institute of Occupational Health, Oulu, Finland Grant no. 50621, 54231 and ERDF European Regional Development Fund Grant no. 539/2010 A31592. | Data is available from the Northern Finland Birth Cohort (NFBC) for researchers who meet the criteria for accessing confidential data. Please, contact NFBC project center (NFBCprojectcenter@oulu.fi) and visit the cohort website (www.oulu.fi/nfbc) for more information |
| NFBC1986 | We thank all cohort members and researchers who have participated in the study. We also wish to acknowledge the work of the NFBC project center. | NFBC1986 received financial support: EU QLG1-CT-2000-01643 (EUROBLCS) Grant no. E51560, NorFA Grant no. 731, 20056, 30167, USA / NIHH 2000 G DF682 Grant no. 50945. | Data is available from the Northern Finland Birth Cohort (NFBC) for researchers who meet the criteria for accessing confidential data. Please, contact NFBC project center (NFBCprojectcenter@oulu.fi) and visit the cohort website (www.oulu.fi/nfbc) for more information. |
| NOMA | We would like to thank the intervention teams at Department of Nutrition, University of Oslo and at Akershus University College of Applied Sciences (OsloMet). We also want to thank Inger Ottestad, Lena Leder, Jacob Juel Christensen, and Lene Frost Andersen, all from the University of Oslo, Department for Nutrition, for their expertise in clinical nutrition, gene expression analysis and dietary assessment methods during the intervention. We would also like to thank all the participants in the study. | This study was supported by Akershus University College of Applied Sciences, Norway, the University of Oslo, Norway, the Throne-Holst Foundation for Nutrition Research, Oslo, Norway, Nordplus Horizontal (NPHZ-2013/10151) and Mills DA, Oslo, Norway. | Any researcher interested in this study should contact Stine M. Ulven (smulven@medisin.uio.no) and visit the website (https://www.med.uio.no/imb/english/research/projects/noma/index.html) for more information |
| OULU1935 | We thank all cohort members and researchers who participated in the study. We also wish to acknowledge the work of the NFBC project center. |  | Data is available from the Northern Finland Birth Cohort (NFBC) for researchers who meet the criteria for accessing confidential data. Please, contact NFBC project center (NFBCprojectcenter@oulu.fi)and visit the cohort website (www.oulu.fi/nfbc) for more information. |
| OULU1945 | We thank all cohort members and researchers who participated in the study. We also wish to acknowledge the work of the NFBC project center. |  | Data is available from the Northern Finland Birth Cohort (NFBC) for researchers who meet the criteria for accessing confidential data. Please, contact NFBC project center (NFBCprojectcenter@oulu.fi) and visit the cohort website (www.oulu.fi/nfbc) for more information. |
| PANIC | We are grateful to all children and their parents and caregivers who have participated in the PANIC Study. We are also indebted to all members of the PANIC research team for their invaluable contribution in the acquisition of the data throughout the study. | The PANIC study has been supported by grants from Ministry of Education and Culture of Finland, Ministry of Social Affairs and Health of Finland, Research Committee of the Kuopio University Hospital Catchment Area (State Research Funding), Finnish Innovation Fund Sitra, Social Insurance Institution of Finland, Finnish Cultural Foundation, Foundation for Paediatric Research, Diabetes Research Foundation in Finland, Finnish Foundation for Cardiovascular Research, Juho Vainio Foundation, Paavo Nurmi Foundation, Yrjö Jahnsson Foundation, and the city of Kuopio. | The PANIC data are available for researchers who meet the criteria for accessing confidential data and have signed the agreement for access to the data. Decisions on giving access to the data are made in the management team of the study on a monthly basis. Information about the PANIC study is available on the webpages of the study www.panicstudy.fi/en/etusivu  and from the Principal Investigator of the study (timo.lakka@uef.fi). |
| RCGP-RSC | We would like to thank the participating practices and patients for providing the data for this cohort. | The development of the RCGP RSC data set is supported by surveillance work funded by Public Health England. | To work with RCGP RSC data should download and complete data request form [Word] avaliable on the website https://www.rcgp.org.uk/clinical-and-research/our-programmes/research-and-surveillance-centre.aspx and send it to MedicalDirectorRSC@rcgp.org.uk |
| Rotterdam Study | We are grateful to the study participants, the staff from the Rotterdam Study, and the participating general practitioners and pharmacists. | The Rotterdam Study is funded by Erasmus MC and Erasmus University, Rotterdam, the Netherlands; the Netherlands Organisation for Scientific Research; the Netherlands Organisation for the Health Research and Development; the Research Institute for Diseases in the Elderly; the Ministry of Education, Culture and Science; the Ministry for Health, Welfare and Sports; the European Commission, and the Municipality of Rotterdam. | Data can be obtained on request. Requests should be directed toward the management team of the Rotterdam Study (secretariat.epi@erasmusmc.nl), which has a protocol for approving data requests. Because of restrictions based on privacy regulations and informed consent of the participants, data cannot be made freely available in a public repository. |
| SYSDIMET/ HEALTGRAIN | We thank the laboratory technologists T. Onnukka  and K. Kettunen from the Department of Clinical Nutrition, University of Eastern Finland, Kuopio, Finland, and G. Trischler from the Department of Internal Medicine II-Cardiology, University of Ulm Medical Center, Ulm, Germany for their skilful work. | This study was supported by: the Academy of  Finland (117844 and 118590 [to M. Uusitupa]; 131460 [to K. Poutanen]; 130469 [to H. Mykkänen] and 131593 [to V. D. F. de Mello]); the Kuopio University Hospital (5106, 5168, 5254 [to M. Uusitupa]); the Finnish Diabetes Research Foundation; the Sigrid  Juselius Foundation; the Nordic Centre of Excellence on ‘Systems biology in controlled dietary interventions and cohort studies’  (SYSDIET; 070014); and the European Commission in the Communities  6th Framework Programme, Project HEALTHGRAIN (FOODCT-2005-514008). | Any researcher interested in studying the Sysdimet/HealthGrain data should contact the researchers. Please send an email to marjukka.kolehmainen@uef.fi |
| UK Biobank | We would like to thank all the participants of UK Biobank for their vital contribution to the resource. | UK Biobank is funded by the Medical Research Council, Wellcome Trust, Department of Health, Scottish Government, the Welsh Assembly Government, British Heart Foundation, Cancer Research UK and the Northwest Regional Development Agency. | All bona fide researchers can apply to use the UK Biobank resource after registration on the Access Management System https://bbams.ndph.ox.ac.uk/ams/.  For more information you could visit website www.ukbiobank.ac.uk  or contact the access team: access@ukbiobank.ac.uk |
| other | In addition we acknowledge funding to collaborators in EUCAN-connect, a federated FAIR platform enabling large-scale analysis of high-value cohort data connecting Europe and Canada in personalized health (H2020 No 824989), as well as many volunteers who contributed to DataSHIELD (http://datashield.ac.uk/) and MOLGENIS (http://molgenis.org) open source projects used in this project. |  |  |
