# Supplementary figures and images for: LongITools: Dynamic longitudinal exposome trajectories in cardiovascular and metabolic noncommunicable diseases
Source: Environ Epidemiol. 2021 Dec 28;6(1):e184. doi: 10.1097/EE9.0000000000000184 (PMC8835657; doi:10.1097/EE9.0000000000000184)

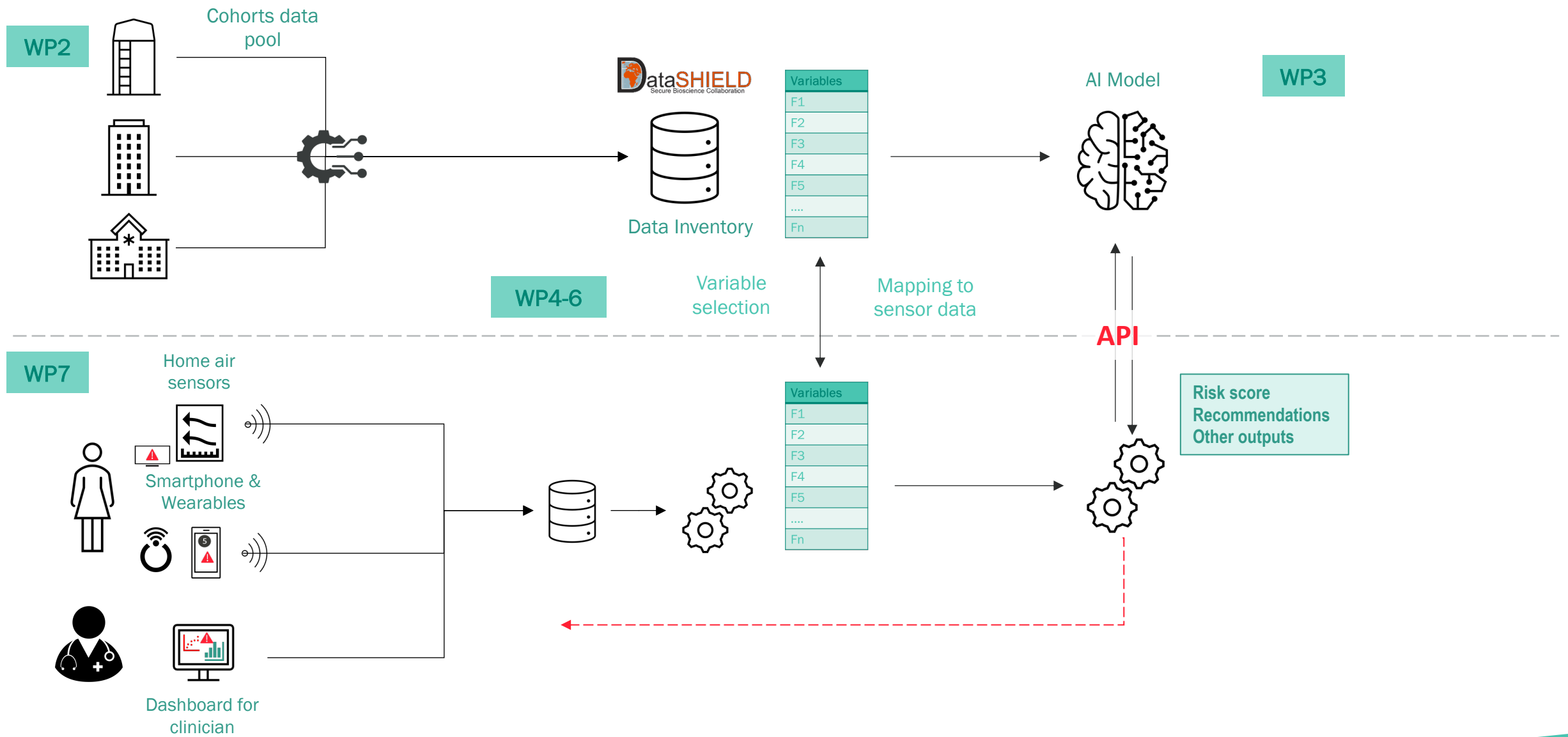

Supplement: Supplementary file 2 [file ee9-6-e184-s002.pdf]
